# Fear Engrams are Formed in the Locus Coeruleus under Contextual Fear Conditioning

**DOI:** 10.17912/micropub.biology.002283

**Published:** 2026-10-26

**Authors:** Mako Shimizu, Amy Jo Stavnezer

**Affiliations:** 1 Neuroscience Program, College of Wooster, Wooster, OH, United States; 2 Neuroscience Program, Psychology Department, College of Wooster, Wooster, OH, United States

## Abstract

Fear memory, a vital survival mechanism, is closely linked to posttraumatic stress disorder (PTSD). PTSD results in persistent negative emotional symptoms following a traumatic experience and is currently difficult to treat. Traditionally associated with the hippocampus and amygdala, recent studies have drawn attention to the locus coeruleus (LC) as an indirect modulator of arousal and stress responses. Using contextual fear conditioning and immunohistochemistry, we found that fear engrams are formed directly in the LC in the TRAP2 mouse model. Therefore, the LC appears to serve as more than an indirect modulator of fear memory, opening new strategies for treating PTSD.

**Figure 1. Identification of fear engrams in the LC during CFC:** (A) Overview of the biological stages of memory processing, including encoding, consolidation, retrieval, and reconsolidation or extinction following re-exposure. (B) Neuronal circuit underlying fear conditioning and its extinction. The red line is associated with the promotion of fear response, while the blue line is for its inhibition. This figure was created based on the conceptual framework described in Kaplan et al. (2011). (C) Experimental design for contextual fear conditioning and TRAP labeling. Mice in the experimental group received three foot shocks (1 s, 0.8 mA) at 120, 180, and 240 seconds after placement into the chamber, while the control group did not. All mice received intraperitoneal 4-OHT injection (55 mg/kg) immediately after training to induce ZsGreen expression in neurons active during memory formation. Animals were kept in darkness for 72 h, returned to a 12-h light/dark cycle for two days, underwent memory testing, and were sacrificed 90 min later for Fos IHC. (D) Schematic of activity-dependent TRAP labeling. Neuronal activity induces expression of IEG (Fos) promoter-driven CreERT2, which remains in the cytoplasm until TM or 4-OHT is administered. Upon 4-OHT treatment, CreERT2 translocates to the nucleus, where it mediates recombination of a loxP-controlled reporter transgene, resulting in permanent expression of ZsGreen. Adapted from Allen et al., 2017; DeNardo et al., 2019; Guenthner et al., 2013. (E) Example of LC quantification. (i) The LC boundary was defined around the TH+ cells (red), incorporating the position of LC-GABAergic neurons based on Breton-Provencher & Sur, 2019. (ii) The ZsGreen+ cells (green) and ZsGreen+/Fost double-positive cells representing engrams (overlapping of green and blue (sky blue); indicated by yellow arrows) were quantified in the LC. (F) Representative LC cell counting in control and experimental groups. (G) Behavioral and cellular outcomes of the main experiment. The experimental group exhibited significantly higher freezing behavior compared with controls (p < 0.006) and showed greater overlap between neurons activated during training and testing phases (p < 0.007), indicating enhanced engram reactivation. (H) Proposed model of vagus nerve stimulation (VNS) effects. Activation of LC extinction engrams in the direct pathway and higher brain modification in the indirect pathway may suppress the fear response caused by the activation of the LC original fear engrams.

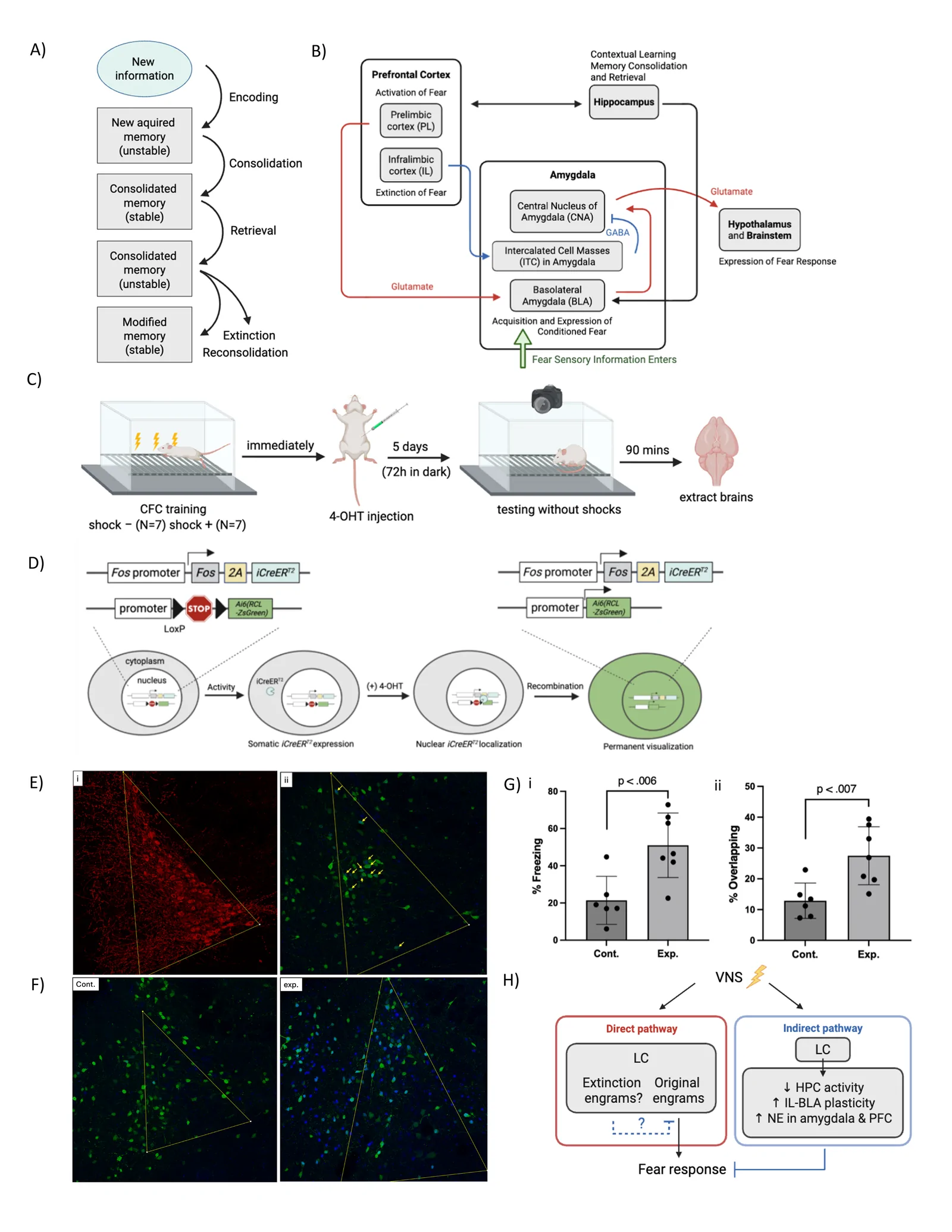



## Description

Post-traumatic stress disorder (PTSD), affecting 3.9% of the global population, is an anxiety disorder caused by a traumatic event, leading to flashbacks, hypervigilance, and social avoidance (Bisson, 2007; Koenen et al., 2017). Contextual fear memory refers to the memory of a particular environment where a fear-inducing event occurred. Individuals with PTSD struggle to suppress activation of fear memories associated with a traumatic event, leading to inappropriate and persistent fear responses. Consequently, when the individual later returns to a similar context, this memory can cause a fear response even in the absence of the original fear-inducing stimulus. Through evolution, organisms have developed this memory as an essential ability for survival, allowing them to respond appropriately in threatening situations (Lee & Kaang, 2023).

Memory is processed by underlying biological processes, including encoding, consolidation, retrieval, reconsolidation, and extinction ( Figure 1A ). Encoding is the first stage, where sensory input is converted into neuronal signals for short-term memory storage (Asuncion & Al Khalili, 2026). This is followed by the consolidation stage, where these signals are stabilized, and long-term memories begin to be created. In consolidation, synaptic connections of memory traces are strengthened by increasing glutamate receptor sensitivity, calcium influx, and some gene expression activated by the transcription factor called c-AMP-response element-binding protein (CREB; Arihara et al., 2023; Willard & Koochekpour, 2013).

Retrieval is the process of accessing stored information by reactivating the neuronal pathway. Interestingly, this reactivation makes the memory temporarily unstable due to protein degradation in the hippocampus, allowing it to be modified, strengthened, or weakened (Lee et al., 2008). This process not only induces memory extinction but also reconsolidation. At the point of retrieval, if the re-exposure to the conditioned stimulus is brief and there is either a prediction or a mismatch between expectation and outcome, the memory trace can enter the reconsolidation process and be restabilized through a second round of synaptic remodeling. In contrast, if re-exposure to the conditioned stimulus is extended but the unconditioned stimulus is not present, meaning the expected outcome does not occur for a long time, memory extinction can occur (Kida, 2020). During extinction, the memory can be weakened by suppressing the original memory trace and generating a new memory trace that inhibits the prior association (Arihara et al., 2023; Lee & Kaang, 2023). Understanding these biological stages is crucial for exploring how the brain stores and modifies memories.

In terms of PTSD, current treatments primarily rely on extinction-based therapies, where patients are repeatedly exposed to trauma-related stimuli in a safe environment to disconnect the traumatic event from fear. However, symptoms persist in approximately 40–60% of patients, highlighting the need for more effective therapeutic strategies (Bradley et al., 2025). Advancing our understanding of molecular mechanisms involved in contextual fear memory is essential for developing improved therapeutic strategies for PTSD.

Several brain regions form the neural circuit responsible for fear memory ( Figure 1B ). The amygdala is central to processing emotions, such as fear, aggression, and pleasure, and linking emotions to memories (AbuHasan et al., 2025). In the fear conditioning process, the amygdala acts as a warning system, detecting danger and initiating defensive responses. The hippocampus is primarily responsible for the learning and memory processes, especially encoding, consolidation, and retrieval related to temporal and contextual information (Dejean et al., 2015). Lastly, the prefrontal cortex (PFC) involves both activation of fear and suppression of the original memory and regulates the fear response (Dejean et al., 2015; Kaplan et al., 2011).

To generate a fear response, sensory information is transmitted from the basolateral amygdala (BLA) to the hypothalamus and brainstem through the central nucleus of the amygdala (CNA) excitatory glutamate projection. Within the PFC, the prelimbic (PL) region promotes fear expression through excitatory glutamate projections to the BLA, whereas the infralimbic (IL) region supports extinction by engaging GABAergic intercalated cell masses (ITC) to inhibit CNA output. Therefore, the dynamic balance between PL-mediated excitation and IL-mediated inhibition enables the PFC to modulate amygdala function and regulate fear expression. The hippocampus communicates with both the amygdala and PFC to integrate spatial and temporal context in fear processing (Kaplan et al., 2011).

Additional signals from the brain–gut axis could modulate contextual fear memory through the Locus Coeruleus (LC), a critical norepinephrine source in the dorsal pons. The vagus nerve is the main parasympathetic component of this axis, with vagal afferents projecting to the nucleus tractus solitarii (NTS) in the dorsomedial medulla, which integrates these signals and relays them to the LC (Breit et al., 2018). Following this, LC neurons project to the amygdala and hippocampus to modulate emotional and episodic memory, and to the PFC to control attention and higher-order cognitive processes such as decision-making (Bari et al., 2020; Breit et al., 2018; Olsen et al., 2023).

In terms of PTSD, vagus nerve stimulation (VNS) has emerged as a new therapeutic approach ( Figure 1H ). One of the most consistently reported neurophysiological effects of VNS is a reduction in hippocampal activity, potentially mediated by enhanced GABAergic signaling (Breit et al., 2018; Capone et al., 2015). Also, a previous study showed that VNS paired with extinction learning facilitates synaptic plasticity between the IL and the BLA, suppressing the downstream signaling to express a fear response (Peña et al., 2014). Finally, the enhancement of extinction learning induced by VNS is thought to be mediated by the norepinephrine (NE) infusion into the amygdala, which facilitates consolidation of extinction memory, and also increases NE levels in the mPFC, which could strengthen the extinction process (Berlau & McGaugh, 2006; Mueller et al., 2008). Elevated neuronal activity in the LC has been observed during memory consolidation, reconsolidation, and extinction following CFC (Arihara et al., 2023). Given its role as an intermediate between the VN and higher-order brain regions, the LC is thought to be a critical component of the neural circuit through which VNS facilitates fear extinction.

One approach for investigating memory in the brain is through engrams, neuronal populations activated during encoding and later reactivated by related stimuli during retrieval (Josselyn & Tonegawa, 2020). These populations of cells undergo physical and chemical changes during memory processing and have been proven to be necessary and sufficient for fear memory (Lee & Kaang, 2023; Liu et al., 2012). Following CFC training, engrams were identified in 117 of 247 rodent brain regions, including the hippocampus, amygdala, PFC, and brainstem regions, but the LC was not specifically examined (Roy et al., 2022). Therefore, though LC is active during several stages of memory processing following CFC, the overlapping expression representing engrams specifically in the LC has not been explored (Arihara et al., 2023).

To determine whether LC engrams exist in fear memory storage, we performed CFC in TRAP2 mice to induce contextual fear memory. A single memory engram can contain functionally distinct neuronal ensembles, Fos- and Npas4-dependent, that respectively promote memory generalization and discrimination, which differentially regulate memory expression (Sun et al., 2020). In the present study, we focused on the Fos-dependent neuronal population in both memory encoding and retrieval and identified putative Fos-dependent engram cells. We separately labeled neurons active during memory encoding by administering 4-OHT immediately after CFC training to induce TRAP2-mediated labeling of Fos expression, and neurons active during retrieval by immunohistochemistry for Fos following the CFC test ( Figure 1C ). We then analyzed the overlap between these two neuronal populations to identify putative engram cells

After successfully inducing conditioned fear, as demonstrated by increased freezing in the experimental condition ( Figure 1G i), we observed a significant increase in engram number in the LC of fear-conditioned mice compared with controls ( Figure 1F, Gii). Traditionally, the LC was considered to modulate memory processes by influencing higher-order brain regions; we have now localized engrams associated with contextual fear memory in the LC. This supports the hypothesis that the LC participates in the direct storage of contextual fear memory and could greatly advance our understanding of the large-scale engram networks and mechanisms underlying contextual fear memory.

Though the present study did not directly examine VNS or extinction learning, and therefore cannot establish a direct relationship between LC engrams and VNS-mediated therapeutic effects, we know VNS can modulate fear memory. In addition, a previous study showed that LC activity is necessary for therapeutic effects of VNS on PTSD-like symptoms in mice (Calderon-Williams et al., 2024). Therefore, future research could explore how these LC engrams are associated with memory extinction under VNS. In addition, research could determine if these engrams are extinction engrams, identified in the hippocampus and amygdala, and have been proposed to suppress the activation of original fear-memory engrams (Herry et al., 2008; Lacagnina et al., 2019), and if they are enhanced with VNS. Lastly, it is necessary to explore the functional connectivity and activity of engram populations across distributed brain networks, targeting the interaction between the LC and higher-order regions. These studies may facilitate the development of more effective PTSD treatments, particularly for those who do not respond to current extinction-based therapies.

## Methods

**Mice**

We used 13–15 week-old male and female Fos2A/Ai6 (TRAP2) mice homozygous for Fos ^(2A-iCreERT2)^ and Gt(ROSA)26Sor ^tm6(CAG-ZsGreen1)Hze^ /J (Ai6(RCL-ZsGreen) (DeNardo et al., 2019; Guenthner et al., 2013). TRAP2 is a genetic approach that enables the permanent labeling of neurons that are active during a specific time window, providing a powerful tool for identifying immediate early gene expression (IEG), which ultimately influences behavior (Arihara et al., 2023; Reijmers et al., 2007). In neurons, IEGs expression (including Fos) is triggered by external stimuli, including neurotransmitter release, membrane electrical activity, and neurotrophic growth factors induced by behavioral training, as well as physiological stimuli (Sheng & Greenberg, 1990). Mice were housed in cages of 2-4 mice, maintained on a 12 h light/dark cycle (8 AM/8 PM), and had access to food and water *ad libitum* . In total, 18 mice (9 male, 9 female) were included in this study. Four mice were used for two pilot experiments (2 each), and the remaining 14 mice were utilized in the main experiment, assigned equally to the control group and experimental group. Given that LC involvement in CFC is still a new research area, we first confirmed that Fos was expressed in LC after training and after testing, respectively (pilot study data, not reported).

**Contextual Fear Conditioning Task**

CFC was performed to induce contextual fear memory ( Figure 1C ). Prior to conditioning, mice were transported daily to a holding room for 1 hour and handled for 2–3 minutes per day for 3 days. On the day of CFC training, mice were again transported to the holding room for one hour before training. All behavioral training and testing took place in Columbus Instruments PACS conditioning chambers, constructed with four black plastic walls, a transparent door ceiling, and a stainless-steel rod floor connected to a shock generator. During conditioning, each mouse was placed into the chamber for a total of 4.5 minutes. Mice in the experimental group received three foot shocks (1 s, 0.8 mA) at 120, 180, and 240 seconds after placement into the chamber and returned to their home cages 30 seconds after the final shock. Control mice remained in the chamber for the same total duration without shock exposure. The chamber was cleaned with 70% ethanol between mice.

**Neuronal Tagging**

Recombination of the ZsGreen transgene was induced by an intraperitoneal injection of tamoxifen or 4-hydroxytamoxifen (4-OHT; 55 mg/kg), given immediately after the contextual fear conditioning. In this system, when a neuron is activated in the presence of tamoxifen (TM), an enzyme called CreER ^T2^ enters the nucleus, where it mediates recombination of transgenes, resulting in permanent expression of the reporter protein ( Figure 1D ). Following the 4-OHT injection, mice were placed back in the home cage and placed in the dark housing room for 72 hours to minimize non-specific neuronal tagging (Denny et al., 2014). Then, mice were placed back in the colony with a 12 h light/dark cycle for two days before retention testing.

Testing occurred 5 days after the conditioning to assess recall of the contextual fear memory. Mice in both control and experimental groups were exposed to the original conditioning context for 5 minutes without shocks. Mice were returned to the colony room for 90 minutes following the completion of testing until tissue collection. All behavior was recorded with a video camera mounted on the top of the chamber, and freezing behavior was analyzed to assess conditioned fear by counting the number of seconds the animal was immobile except for breathing.

**Tissue Collection**

Ninety minutes after retention testing, mice were euthanized for brain collection. Mice were deeply anesthetized with 5% isoflurane and decapitated to extract their brains. Brains were immediately fixed in 4% paraformaldehyde (PFA) for 24 hours and then cryoprotected in 20-30% sucrose for three days. Finally, brains were coronally cryo-sectioned at 35 µm and stored in cryoprotectant at -20 °C until staining.

**Immunohistochemistry**

For each mouse **,** 6 free-floating coronal sections encompassing the LC were washed in 1x PBST (10 mins x 3) and blocked for 90 mins in 5% normal donkey serum (NDS) in 1x PBS with 0.25% Triton X-100 (PBST). Tissue was then incubated overnight at room temperature in 1:3000 chicken anti-Fos (SYSY, 226009) and 1:1000 rabbit anti-tyrosine hydroxylase (TH) primary antibodies (Novus Biologicals, NB300-109) diluted in 5% NDS/PBST. The following day, tissues were washed in PBS (10 mins x 3) and blocked in 5% NDS/PBST for 1h, then incubated in 1:2000 Alexa Fluor 647-donkey anti-chicken (Invitrogen, A-78952) and 1:1000 Alexa Fluor 555-donkey anti-rabbit secondary antibodies (Invitrogen, A-31572) in 5% NDS/PBST for 90 mins. After secondary incubation, sections were washed again with PBS (10 mins x 3), counterstained with DAPI (1:1000, ThermoFisher), washed with PBS (10 mins x 3), and mounted on glass slides using Antifade Mounting Medium (Vectashield, H-1000).

**Imaging and Quantification**

For each mouse, 8-12 LC sections, across both hemispheres, were imaged using a confocal microscope (Olympus Fluoview FV3000). Those sections were located around interannual –1.54 mm to –2.00 mm, and 6 sections around –1.80 mm were randomly chosen for comprehensive LC cell counting (Breton-Provencher & Sur, 2019).

The LC region was defined based on the location of TH+ cells in each group. Given that GABAergic neurons also exist in the LC, the anatomical boundary was drawn based on the TH+ cell population, incorporating the location of the GABAergic neurons based on Breton-Provencher & Sur, 2019 ( Figure 1E i). To minimize bias in determining LC boundaries, only the TH channel was viewed during this process. Next, the ZsGreen+ cells and double-positive (Fos+/ZsGreen+) cells were quantified in the LC ( Figure 1E ii, F). All quantification was conducted using Fiji.

**Statistical Analysis**

To assess the presence of putative engram cells, the ratio of double-labeled cells (Fos+/ZsGreen+) relative to the total ZsGreen+ population was calculated to generate a reactivation ratio. A higher proportion of reactivated cells in the experimental compared to the control group indicates the existence of engrams supporting contextual fear memory. Statistical comparison of reactivation ratios between groups was performed using a t-test, with significance defined as p < 0.05 (Fig 1Gii).
